# Hospitalization costs for community-acquired pneumonia in Dutch elderly: an observational study

**DOI:** 10.1186/s12879-016-1783-9

**Published:** 2016-09-02

**Authors:** Conrad E. Vissink, Susanne M. Huijts, G. Ardine de Wit, Marc J. M. Bonten, Marie-Josée J. Mangen

**Affiliations:** 1 Julius Center for Health Sciences and Primary Care, University Medical Center Utrecht, Utrecht, The Netherlands; 2 Present address: Department of Psychiatry, University Medical Center Utrecht, A01.126, Heidelberglaan 100, Utrecht, 3508 GA The Netherlands; 3 Department of Respiratory Medicine, University Medical Center Utrecht, Utrecht, The Netherlands; 4 Department of Medical Microbiology, University Medical Center Utrecht, Utrecht, The Netherlands

**Keywords:** Community-acquired pneumonia, Costing study, Elderly, Hospitalization, Invasive pneumococcal disease, Risk-groups

## Abstract

**Background:**

Community-acquired pneumonia (CAP) is one of the most common infections, especially in the elderly (≥65 years). The aim of this study was to quantify hospitalization costs for CAP in different age groups and in patients with different CAP risk profiles. Secondary objectives were to assess disease severity differences between placebo and vaccine receiving participants and identify cost driving factors of CAP in hospitalized elderly in the Netherlands.

**Methods:**

This prospective cohort study of hospitalized CAP patients was executed in parallel to the Community Acquired Pneumonia Immunization Trial in Adults (CAPiTA). Within the CAPiTA, a cohort of 84,496 subjects aged ≥65, all suspected CAP-episodes presenting in one of the 58 participating hospitals between September 2008 and August 2013 were included. CAP was diagnosed on clinical and radiographical criteria. Invasive pneumococcal disease (IPD) and non-IPD-CAP episodes, regardless of the causing pathogen, were evaluated separately. Costs were calculated by multiplying recorded healthcare resources with Dutch unit cost prices for the year 2012. Multivariate regression analysis was performed to identify cost drivers.

**Results:**

In the sentinel hospitals 3225 suspected CAP and IPD episodes were included, of which 1933 were radiographically confirmed by chest X-ray. Analyses were conducted on confirmed CAP episodes only. Overall mean length of hospital stay was 12.1 days, the in-hospital mortality rate was 11.26 %, and mean costs were €8301 (95 % CI: €7760–€8999). When stratified in age-categories 65–74, 75–84 and ≥85, mean hospitalization costs were €8674, €8770 and €6197, respectively (*p* = 0.649). IPD-CAP and non-IPD-CAP mean hospitalization costs were €13,611 and €8081, respectively. Higher CURB-65 score and individuals at medium risk for developing pneumococcal disease were significantly associated with higher costs. Being male, lower age, previous admissions, lower risk, lower urbanity and higher socio-economic status were associated with lower costs.

**Conclusions:**

Mean hospitalization costs of a CAP subject were €8301 and higher for IPD-CAP compared to non-IPD-CAP cases. Medium risk patients and higher CURB-65 scores were identified as cost driving factors.

**Electronic supplementary material:**

The online version of this article (doi:10.1186/s12879-016-1783-9) contains supplementary material, which is available to authorized users.

## Background

Community-acquired pneumonia (CAP) is one of the most common infectious diseases in the world [1–4]. In Europe, the incidence of all-cause CAP (hospitalized and outpatient) in the elderly population (≥65 years) is estimated at 14 per 1000 person years [5, 6]. Approximately 56 % (equal to €5.7 billion) of CAPs economic burden in Europe is ascribed to hospitalization expenses [7]. The other costs can be attributed to outpatient care, loss of workdays and drug expenses. Increasing CAP incidence due to growth of the elderly population in the upcoming years [8–11], more severe episodes due to increasing comorbidity within the aging population [12], and limited economic resources demand for new preventive measures [13]. In Western countries like the Netherlands, new healthcare interventions will be evaluated for their costs and effects and detailed cost estimates are indispensable in this process. However, most larger studies describing CAP costs were conducted retrospectively and based on insurance claims with limited clinical information [4, 14–19]. The few prospective studies on CAP hospitalization costs covered relative small cohorts only [20–22].

The Community Acquired Pneumonia Immunization Trial in Adults trial (CAPiTA), a nation-wide study assessing the efficacy of the 13-valent pneumococcal vaccine (PCV13) in the elderly, created a unique opportunity for quantifying hospitalization costs of CAP [23, 24]. A parallel study was designed to estimate costs for hospitalized CAP patients, including patients with and without invasive pneumococcal disease (IPD), stratified by age- and risk-groups for developing pneumococcal infections and by outcome (i.e. mortality). Secondary objectives were to analyze disease severity differences between PCV13 and placebo cohorts and to identify cost drivers in hospitalized CAP cases.

## Methods

### Study subjects

The etiology and prognosis of community-acquired pneumonia (Etio-CAP) study is a prospective cohort study conducted in parallel to CAPiTA [23, 24]. In this double blind randomized clinical vaccination trial in the Netherlands, the efficacy of PCV13 in adults was evaluated by vaccinating 84,496 subjects of ≥65 years of age with either placebo or PCV13 with an average follow-up period of 3.97 years [24]. Detailed information, in- and exclusion criteria and outcome measures have been previously described [23, 24]. Follow-up was organized in 59 sentinel centers (58 hospitals and 1 outpatient clinic) between September 2008 and August 2013, where the 58 hospitals participated in the Etio-CAP study. When a CAPiTA-participant was registered to one of these hospitals with a suspicion of pneumonia, regardless of the causing pathogen, clinical data were collected according to the CAPiTA protocol. When admitted, additional data, like use of resources and complications, were collected as part of the Etio-CAP-study.

### Definitions

CAP was defined, regardless of the causing pathogen, as the presence of two or more clinical signs of pneumonia together with a chest x-ray consistent with pneumonia [23]. IPD was defined as *Streptococcus pneumoniae* cultured from a sterile site (e.g. blood). The analysis was conducted for three groups: ‘total CAP’ (i.e. all confirmed CAP cases), non-IPD-CAP (i.e. confirmed CAP cases, no IPD) and ‘IPD-CAP’ (i.e. cases with confirmed CAP and IPD). Subjects with IPD without CAP were not included in the analyses.

Admissions within 30 days post-discharge of a suspected CAP hospitalization episode were labeled as a readmission and treated as a continuation of the previous admission with regard to a) diagnosis (CAP or IPD) and b) costs (i.e. resources used in first and following admissions, and associated costs were added up).

The study was approved by the Central Committee on Research Involving Human Subjects (CCMO11.0810/TV/NL23014) as a sub-study of the CAPiTA-trial. Therefore no separate informed consent was required for the Etio-CAP study.

### Data collection

Data collected included length of stay (LOS) and intensive care unit (ICU) admission, comorbidities at moment of admission (see Additional file 1: Table S1), date of symptom onset and date of symptom relieve. Based on the presence of comorbidities, subjects were stratified into high (i.e. immunocompromised patients), medium (i.e. presence of other chronic conditions) and low risk for pneumococcal infections (Additional file 1: Table S1).

Data included for the Etio-CAP-study concerned: mortality (with minimum follow-up of 6 months after hospital admission, i.e. confirmation of vital status by the GP of the participant), laboratory results, number of hospital admissions 1 year prior to index CAP hospitalization episode, diagnostic and therapeutic interventions for both pneumonia and its complications, additional “waiting days” in the hospital (i.e. before home care could be organized), and location of discharge (own home or nursing home). Pneumonia severity was assessed using the Pneumonia severity index (PSI), as described by Fine et al. [25], and CURB-65 [26]. PSI score was categorized into low (score 1 and 2), medium (score 3 and 4) and high (score 5). Based on postal codes, information on socioeconomic status (continues variable from low to high, scores ranging from -5.5 to 3, with lower scores reflecting lower socio-economic status) and urbanity of neighborhood (from high to low; score 1, 2, 3, 4 and 5) was obtained from Statistics Netherlands and referred to the situation in 2010 [27].

### Costs

Hospitalization costs were calculated by multiplying recorded units of healthcare resources used with corresponding unit prices (see Additional file 1: Table S2) [28]. Unit costs were either taken from the Dutch manual for healthcare costing research [28, 29] or were calculated from a unit price medium of different hospital price lists available. All costs were expressed for the year 2012, and if necessary, updated using Dutch consumer price indices [30].

### Statistical Analysis

Data were analyzed using IBM SPSS Statistics for Windows, Version 20.0. The one-way ANOVA test, Mann-Whitney U-test and the Kruskal-Wallis test were used to compare quantitative variables and the Chi-square test was used for categorical variables. Intubation days at the ICU were recorded in a dichotome (yes or no) and a quantitative way (e.g. number of days of intubation). There were no missing data with the exception of number of intubated ICU days (0.6 %) and number of previous admissions (0.2 %). Missing data were imputated using multiple imputation. A stepwise multiple regression model was used to identify cost drivers with log-transformed hospitalization costs as a dependent variable. Explanatory variables were gender, age, previous admission (categorized as: none, one, two, three or more admissions), smoking status (non-smoker, previous smoker, current smoker), risk-level, socioeconomic status and urbanity, and CURB-65. The latter was used instead of PSI scoring, because of overlapping definitions between PSI and risk levels as both are estimated using comorbidity information. The 95 % confidence interval (CI) was determined using bootstrapping with 1000 iterations. A *p*-value of <0.05 was considered significant.

## Results

### Study participants

**Fig. 1:**
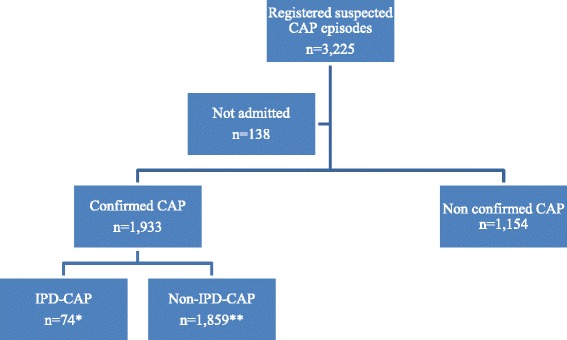
Flowchart of the study. Legend: *Including 3 readmissions; **Including 145 readmissions. Abbreviations: CAP: community-acquired pneumonia; IPD-CAP: invasive pneumococcal disease

There were 3225 registered suspected CAP episodes, of which 138 were not hospitalized. Of these suspected and hospitalized CAPs 1154 cases were not confirmed as CAP (i.e. no positive chest X-rays and/or <2 clinical signs of pneumonia) and consequently excluded from the analysis (Fig. 1). Of the remaining 1933 confirmed CAP admissions, 148 were readmissions, yielding 1785 episodes for analysis. Of these, 74 had IPD-CAP and 1714 had non-IPD-CAP) (Fig. 1).

The majority (73 %) of CAP patients were male, and the mean length of hospitalization was 12.1 days (95 % CI: 2–42 days) consisting of 92 % ward days and 8 % ICU days (Table 1). The 30-day mortality rate of low, medium and high risk-groups was 4, 10.4 and 29.1 %, respectively. Compared to non-IPD-CAP cases, IPD-CAP cases had a longer average duration of stay in hospital (*p* = 0.028) and in ICU (*p* = 0.022) (Table 1).

**Table 1:** Baseline characteristics of study population

	Total CAP	IPD-CAP	non-IPD-CAP	*p*-value
Admission^a^, n	1785	71	1714	
Age in years, mean (SD)	77.4 (6.6)	76 (5.5)	77.5 (6.7)	
Male, n (%)	1307 (73.2)	43 (60.6)	1264 (73.7)	*
ReceivedPCV13, n (%)	861 (48.2)	24 (33.8)	837 (48.8)	*
Risk group for developing pneumococcal disease, n (%)				
• Low	248 (13.9)	18 (25.4)	230 (13.4)	
• Medium	1207 (67.6)	47 (66.2)	1160 (67.7)	
• High	330 (18.5)	6 (8.5)	324 (18.9)	
Length of stay in days, mean (95 % CI)	12.1 (11.6–12.6)	15.3 (12.3–18.9)	12.0 (11.5–12.4)	*
• Ward days, mean (95 % CI)	11.1 (10.7–11.6)	13.0 (10.3–15.8)	11.1 (10.6–11.5)	
• ICU days, mean (95 % CI)	1.0 (0.8–1.2)	2.4 (0.9–4.2)	0.9 (0.7–1.1)	*
• Intubationdays, mean (95 % CI)	0.4 (0.3–0.6)	1.1 (0.3–2.2)	0.4 (0.3–0.6)	*
Illness duration in days^b^, mean (95 % CI)	16.9 (16.3–17.5)^c^	19.8 (16.1–24.1)	16.7 (16.1–17.4)^c^	
Days between onset and hospital admission, mean (95 % CI)	4.9 (4.6–5.2)^d^	4.3 (2.7–6.8)	4.9 (4.6–5.3)^d^	
Pneumococcal severity index (PSI), n (%)				
• I-II	126 (7.1)	5 (7.0)	121 (7.1)	
• III-IV	1327 (74.3)	48 (67.6)	1279 (74.6)	
• V	332 (18.6)	18 (25.4)	314 (18.3)	
In-hospitalmortality, n (%)	201 (11.3)	11 (15.5)	190 (11.1)	
30-day mortality, n (%)	235 (13.2)^e^	11 (15.5)	224 (13.1)^e^	
60-day mortality, n (%)	300 (16.8)^e^	12 (16.9)	288 (16.8)^e^	
6-months mortality, n (%)	444 (24.9)^e^	18 (25.4)	426 (24.9)^e^	
Location of discharge, n (%)	1584 (88.7)	60 (84.5)	1524 (88.9)	
• Home	1371 (86.6)	51 (85.0)	1320 (86.6)	
• Nursing home	160 (10.1)	8 (13.3)	152 (10)	
• Others	53 (3.3)	1 (1.7)	52 (3.4)	

*Statistically significant difference between non-IPD-CAP and IPD-CAP

^a^Admissions within 30 days post-discharge of a suspected CAP hospitalization episode were labeled as a readmission and analyzed as a continuation of the previous admission

^b^Number of days from onset to hospital discharge

^c^11 with missing data

^d^9 with missing data

^e^2 with missing data

### Costs

The mean hospitalization cost for all CAP subjects was €8301 (Median: €4809, 95 % CI: €7760–€8999) (Table 2 and Additional file 1: Table S3). General ward nursing costs accounted for 63 % of total hospitalization costs and ICU nursing for 29 %. When stratified by age groups 65–74, 75–84 and ≥85 years, mean hospitalization costs were €8674, €8770 and €6197, respectively (*p* = 0.649). The mean hospitalization costs per IPD-CAP case were significantly higher than for non-IPD-CAP cases (€13,611 versus €8081, *p* = 0.025).

**Table 2:** Hospitalization costs of CAP and IPD-CAP subjects stratified by age- and risk-groups in 2012 €

Population	Age group (years)	Risk level	N	Mean	95 % Confidence Interval	
					Lower	Upper
Total CAP	All		1,785	8,301	7,760	8,999
	65-74	All	678	8,674	7,841	9,641
		Low	87	8,418	5,870	11,696
		Medium	445	9,118	7,921	10,407
		High	146	7,472	6,163	9,248
	75–84	All	807	8,770	7,748	9,847
		Low	73	10,387	7,222	14,516
		Medium	574	8,270	7187	9,550
		High	160	9,828	7,345	12,811
	≥85	All	300	6,197	5,677	6,764
		Low	42	6,042	4,978	7,188
		Medium	227	6,198	5,606	6,832
		High	31	6,401	5,039	8,238
IPD-CAP	All		71	13,611	8,612	20,434
	65–74	All	31	11,635	6,996	17,982
		Low	8	16,185	5,336	34,137
		Medium	20	10,615	5,831	17,142
		High	3	6,299	3,815	8,960
	75–84	All	34	16,496	7,650	30,360
		Low	6	18,837	3,880	41,063
		Medium	25	16,903	6,182	35,483
		High	3	8,416	1,268	21,697
	≥85	All	6	7,478	3,456	12,416
		Low	2	7,440	3,860	11,020
		Medium	4	7,497	2,238	16,577
		High	0	-	-	-

Due to minimal differences between total CAP and non-IPD-CAP cohorts costs, the latter are presented in Additional file 1: Table S3

**Fig. 2:**
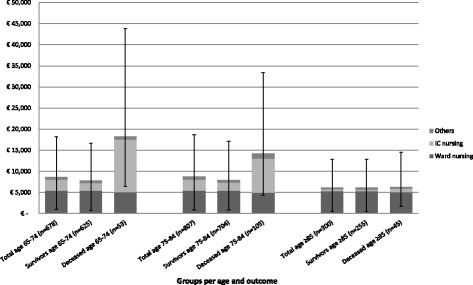
Mean CAP-related hospitalization cost for the total CAP cohort, survivors and deceased persons stratified by age. Error-bars depicting the 95 % confidence interval of the mean. Results obtained using boostrapping

**Fig. 3:**
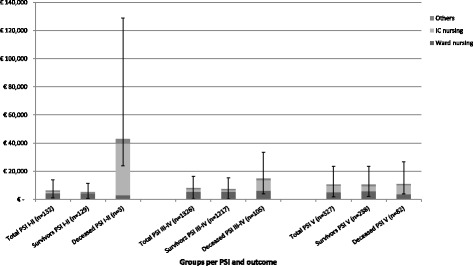
Mean CAP-related hospitalization cost for the total CAP cohort, survivors and deceased persons stratified by severity (i.e. PSI score). Error-bars depicting the 95 % confidence interval of the mean. Results obtained using boostrapping. Abbreviations: CAP: community-acquired pneumonia; PSI: Pneumococcal Severity Index

The mean hospitalization cost for survivors and non-survivors in the total CAP cohort was €7638 and €13,526, respectively (*p* = 0.30) (Figs. 2 and 3). ICU costs of survivors were lower (*p* < 0.001) and ward costs were higher (*p* < 0.001) compared to non-survivors.

Comparison between subjects who received PCV13 (*n* = 861) and placebo (*n* = 924) revealed no differences in LOS (12.03 versus 12.17 days, *p* = 0.919), PSI category (2.13 versus 2.09, *p* = 0.368) and mean hospitalization costs (€8115 versus €8475, *p* = 0.835). Furthermore, LOS, PSI category and costs were similar for subjects with IPD-CAP and non-IPD-CAP.

### Cost drivers

A high CURB-65 score and medium risk profile for pneumococcal CAP were associated with higher hospitalization cost (Table 3). Male gender, increasing age, one or more previous admissions in the past year, low risk profile for pneumococcal CAP, lower urbanity and higher socio-economic status were associated with lower costs.

**Table 3:** Cost driving factors in the CAPcohort

	Multiple regression		
	β	SE	*p*-value
Constant	9.23	0.06	<0.001
Male	−0.06	0.01	<0.001
Age	−0.01	0.00	<0.001
Previous admission	−0.03	0.00	<0.001
CURB-65	0.12	0.01	<0.001
Low risk	−0.09	0.02	<0.001
Medium risk	0.03	0.01	0.049
Urbanity	−0.03	0.00	<0.001
Socioeconomic status	−0.01	0.00	0.02

## Discussion

Among those who participated in the CAPiTA-study, the mean costs of hospitalization for CAP were €8301. Costs for a hospitalized CAP episode with IPD were €13,611, or 168 % of the mean costs for a non-IPD-CAP episode (i.e. €8081). A high clinical severity of disease at the time of hospitalization and having medium risk profile for pneumococcal disease were identified as independent drivers of high costs, whereas male gender, increasing age, previous admission(s) in the past year, low risk profile for pneumococcal CAP, lower urbanity and higher socio-economic status were associated with lower overall costs. Vaccination status was not associated with clinical severity of CAP or costs of hospitalization.

Quantification of costs associated with CAP are usually based on retrospective studies using national insurance claims databases. In a previous retrospective study in Netherlands by Spoorenberg et al. [20], median costs based on ICD recordings were estimated at €3899. In the current study median costs were €4809, and the difference could be explained by the younger cohort in the Spoorenberg et al. study (mean age 63.4 versus 77.4) potentially leading to a shorter LOS (8.5 versus 12 days) and a lower case-fatality rate (30-day mortality: 5.1 versus 13.1 %). Furthermore, in the current study, non-survivor costs were €13,526 and appeared to be 177 % of the hospitalization costs of survivors (i.e. €7638). Also, Spoorenberg et al. did not consider the additional costs of readmissions and included patients of only two general hospitals in the Netherlands, as compared to 58 hospitals, of which seven are tertiary care centers, were included in the current study. Indeed, the proportion of patients needing ICU admission was 21.2 % in tertiary care (24/113) and 12.1 % in general hospitals (202/1672).

Another nationwide ICD-based cost analysis by Rozenbaum et al. [19] reported medium costs of CAP of €6060 and €5937 in those 65–74 and 75–84 years of age, respectively, which is also lower than the medium costs for these age cohorts in the current study (€8674 and €8770, respectively). The study by Rozenbaum et al. was based on reimbursement of hospital expenses, as regulated by Diagnosis Related Groups (in Dutch: Diagnose BehandelCombinatie or DBC). By selecting solely DBCs related to pneumonia, other costs (e.g. treatment of complications) were not considered, possibly explaining the lower costs. Also readmissions were not taken into account, resulting in shorter length of stay in the 65–74 and 75–84 age cohorts (7.7 versus 12.4 and 8.1 versus 12.3, respectively). Furthermore, the use of ICD-coding in both Dutch studies [19, 20] may have caused important misclassification. In another Dutch study across seven Dutch hospitals, the sensitivity of ICD-coding was estimated at 79.5 % [31]. The current prospective study with 1933 patients with confirmed CAP based on standardized diagnostic criteria may, therefore, be more precise and generalizable than previous studies [16, 19–22].

Different settings and healthcare systems makes a comparison between countries nearly impossible at the level of cost estimates [32]. Length of Stay (LOS) has been identified as a major contributor to healthcare resource utilization. We found a mean of 12 days which is within the range of other European studies, [21, 22, 33–40], ranging from 8 up to 13.2 days.

There are also limitations of this study that need to be addressed. An exclusion criterion for the CAPiTA-trial was the presence of immunodeficiency (i.e. individuals at high risk for developing pneumococcal infections), except for patients with asplenia. However, there were 330 (18.5 %) patients with CAP that had developed immune deficiencies after enrollment in the CAPITA-trial. Hospital costs might be slightly underestimated as we did not have exact information on the percentage of patients that received in-hospital rehabilitation services, or because some diagnostic or therapeutic procedures, such as incidental pleural punctures, may not have been reported. Additionally, in the current cohort 10.1 % of the survivors were discharged to a nursing home or rehabilitation center. As information on the total length of stay in nursing home or rehabilitation center was lacking, we could not add these costs to our cost estimates.

## Conclusion

The current study showed variability in hospitalization cost between different ages, disease severities and survival status. The eldest group experienced high mortality and shorter ICU admission. Mean hospitalization cost for a CAP subject was €8301. Costs are significantly higher for IPD-CAP than for non-IPD-CAP cases. With an aging population and a rising life expectancy the disease and economic burden may further increase in the near future.

## Additional files

Additional file 1: Table S1. Comorbidities considered at increased risk for developing pneumococcal infections; Table S2. Unit cost prices; Table S3. Hospitalization costs of non-IPD-CAP subject stratified by age- and risk-category in 2012 €.

## Abbreviations

CAP: Community-acquired pneumonia
CAPiTA: Community Acquired Pneumonia Immunization Trial in Adults
CI: Confidence interval
DBC: Diagnosis Related Groups (in Dutch: Diagnose Behandel Combinatie)
Etio-CAP: Etiology and prognosis of community-acquired pneumonia study
GP: General practice
ICD: International Classification of Diseases
ICU: Intensive care unit
IPD: Invasive pneumococcal disease
IPD-CAP: Community-acquired pneumonia patients with invasive pneumococcal disease
LOS: Length of stay
non-IPD-CAP: Community-acquired pneumonia patient without invasive pneumococcal disease
PCV13: 13-valent pneumococcal vaccine
PSI: Pneumonia Severity Index
