# Erratum to: Hospitalization costs for community-acquired pneumonia in Dutch elderly: an observational study

**DOI:** 10.1186/s12879-016-2015-z

**Published:** 2016-11-24

**Authors:** Conrad E. Vissink, Susanne M. Huijts, G. Ardine de Wit, Marc J. M. Bonten, Marie-Josée J. Mangen

**Affiliations:** 1 Julius Center for Health Sciences and Primary Care, University Medical Center Utrecht, Utrecht, The Netherlands; 2 Present address: Department of Psychiatry, University Medical Center Utrecht, A01.126 Heidelberglaan 100, Utrecht, 3508 GA The Netherlands; 3 Department of Respiratory Medicine, University Medical Center Utrecht, Utrecht, The Netherlands; 4 Department of Medical Microbiology, University Medical Center Utrecht, Utrecht, The Netherlands

## Erratum

In our discussion [1] we erroneously misclassified the study of Spoorenberg et al. [2] as being a retrospective study using ICD records. Readers should know that the study of Spoorenberg et al. [2] is similar to our study, a prospective cohort with confirmed CAP based on standardized diagnostic criteria.

The observed difference in costs per average CAP patients can be partly explained by the younger cohort in the Spoorenberg et al. [2] study (mean age 63.4 versus 77.4) and by the exclusion of immunocompromised patients, patients directly admitted to the intensive care unit and patients having received immunosuppressive therapy in the study of Spoorenberg et al. [2] (in total 439 cases or 24.6% of our study population), leading to a shorter LOS (8.5 versus 12 days) and a lower case-fatality rate (30-day mortality: 5.1 versus 13.1%).
