# Supplementary material for: Hospitalization costs for community-acquired pneumonia in Dutch elderly: an observational study
Source: BMC Infect Dis. 2016 Sep 2;16(1):466. doi: 10.1186/s12879-016-1783-9 (PMC5010739; doi:10.1186/s12879-016-1783-9)
Supplement: Additional file 1: Table S1. — Comorbidities considered at increased risk for developing pneumococcal infections; Table S2. Unit cost prices; Table S3. Hospitalization costs of non-IPD-CAP subject stratified by age- and risk-category in 2012 €. [file 12879_2016_1783_MOESM1_ESM.docx]

**Additional file 1**

**Manuscript**

**Hospitalization costs for community-acquired pneumonia in Dutch elderly: an observational study**

| **Table S1. Comorbidities considered at increased risk for developing pneumococcal infections** | |
| --- | --- |
| Medium | High |
| Alcoholism | AIDS |
| Cerebrospinal fluid leaks | Bronchial obstruction due to primary lung cancer |
| Chronic cardiovascular disease | Functional of anatomic Asplenia |
| Chronic pulmonary disease | Chronic liver disease |
| DM with insulin | Chronic renal failure |
| DM without insulin | Malignancy |
|  | Hodgkin |
|  | Human immunodeficiency virus infection |
|  | Leukemia |
|  | Lymphoma |
|  | Multiple myeloma |
|  | Receipt of immunosuppressive therapy |
|  | Receipt of an organ/bone marrow transplant |

Note: If patients did not meet any of the above criteria they were considered at low risk [1-3].

Abbreviations used: CAP: community-acquired pneumonia; AIDS: acquired immune deficiency syndrome; DM: diabetes mellitus.

| **Table S2. Unit cost prices in 2012 €** | | | | |
| --- | --- | --- | --- | --- |
| Unit | Unit cost (€) |  | Source |  |
| ***Hospital contact*** |  |  |  |  |
| Emergency department visit (per consultation) | €160.34 |  | [4] |  |
| Hospital admission (general ward, per day) | €485.28 |  | [4] |  |
| Intensive care unit (ICU) per day |  |  |  |  |
| without artificial ventilation/day | €1,879.63 |  | [5] |  |
| with artificial ventilation /day | €2,859.81 |  | ^a)^ |  |
| ***Diagnostic procedures, therapy and complications*** | | | |  |
| Blood transfusion | €204.22 |  | [4] |  |
| Bronchoscopy | €483.97 |  | ^a)^ |  |
| Carotid duplex | €63.19 |  | ^a)^ |  |
| Chest X-ray | €96.87 |  | ^a)^ |  |
| Coiling femoral superficial artery | €3,591.82 |  | ^a)^ |  |
| Coronary Angiography | €296.96 |  | ^a)^ |  |
| Coronary stent placement | €721.00 |  | ^e)^ |  |
| CT-angiography of the chest | €244.42 |  | ^a)^ |  |
| CT-scan abdomen | €208.60 |  | ^a)^ |  |
| CT-scan brain | €192.40 |  | ^a)^ |  |
| CT-scan sinus | €192.40 |  | ^a)^ |  |
| CT-scan thorax | €208.60 |  | ^a)^ |  |
| Culture test (e.g. blood, sputum, …) | €21.96 |  | ^a)^ |  |
| Continuous venovenous hemofiltration/day^b^ | €2,961.41 |  | ^a)^ |  |
| Doppler ultrasound | €78.71 |  | ^a)^ |  |
| Echocardiography | €72.95 |  | ^a)^ |  |
| Electric cardioversion | €290.89 |  | ^a)^ |  |
| Electroencelography (EEG) | €94.46 |  | ^a)^ |  |
| Explorative thoracotomy | €6,597.07 |  | ^a)^ |  |
| Heart function test | €85.35 |  | ^a)^ |  |
| Heart valve replacement | €10,185.1 |  | ^a)^ |  |
| Holter monitor | €2,469.74 |  | ^a)^ |  |
| Lung function test | €97.74 |  | ^a)^ |  |
| Lumbar puncture | €316.54 |  | ^a)^ |  |
| Magnetic resonance imaging (MRI) hips^c^ | €244.42 |  | ^a)^ |  |
| Non-invasive artificial ventilation | €291.78 |  | ^a)^ |  |
| Pacemaker placement | €8,426.83 |  | ^a)^ |  |
| Percutaneous coronary intervention | €4,280.64 |  | ^a)^ |  |
| Pericardiocentesis | €51 |  | ^a)^ |  |
| SPECT ^d^ myocard perfusion imaging | €250.87 |  | ^a)^ |  |
| Surgery | €8,427.41 |  | ^a)^ |  |
| Swan-Ganz catheter | €789.35 |  | ^e)^ |  |
| Thoracentesis (drainage) | €231 |  | ^f)^ |  |
| Thoracentesis (only puncture) | €197 |  | ^f)^ |  |
| Transesophageal echocardiography | €87.00 |  | ^a)^ |  |
| Ultrasound, abdomen | €107.15 |  | ^a)^ |  |
| Ultrasound, upper extremity | €192.40 |  | ^a)^ |  |
| Urinary antigen test (e.g. legionella, pneumococcus) | €23.46 |  | ^a)^ |  |
| Video-assisted thoracic surgery (VATS) | €6,761.80 |  | ^a)^ |  |

1. Median costs according to price lists of different hospitals in 2012 (details available on request from first author)
2. Only if occurring during ICU stay and only for the days admitted at ICU.
3. Linked to hospital admissions (e.g. MRI of hip required after failing during admission).
4. Single Photon Emission Computed Tomography
5. Personal communication from the financial unit of the Cardiac and Lung Disease department from UMCU, L. Plug 25 March 2014.
6. Costs were estimated with a bottom up calculation described by Mangen et al.[5].

| **Table S3. Hospitalization costs of non-IPD-CAP subject stratified by age- and risk-category in 2012 €.** | | | | | | | |
| --- | --- | --- | --- | --- | --- | --- | --- |
| Population | Age group | Risk level | N | Mean | 95% Confidence Interval * | | |
|  |  |  |  |  | Lower | Upper | |
| non-IPD-CAP | All |  | 1,714 | 8,081 | 7,522 | 8,687 | |
|  | 65-74 | All | 647 | 8,532 | 7,641 | 9,500 | |
|  |  | Low | 79 | 7,631 | 5,351 | 10,620 | |
|  |  | Medium | 425 | 9,048 | 7,804 | 10,504 | |
|  |  | High | 143 | 7,496 | 6,123 | 9,271 | |
|  | 75-84 | All | 773 | 8,430 | 7,532 | 9,558 | |
|  |  | Low | 67 | 9,630 | 6,505 | 13,888 | |
|  |  | Medium | 549 | 7,877 | 6,921 | 9,055 | |
|  |  | High | 157 | 9,855 | 7,413 | 13,288 | |
|  | ≥85 | All | 294 | 6,171 | 5,663 | 6,699 | |
|  |  | Low | 40 | 5,972 | 4,904 | 7,143 | |
|  |  | Medium | 223 | 6,175 | 5,577 | 6,890 | |
|  |  | High | 31 | 6,401 | 5,067 | 8,176 | |
|  |  |  |  |  |  |  | |
| Legend: * Confidence interval of the mean. | | | | | | | |

**References**

1. van Hoek AJ, Andrews N, Waight PA, Stowe J, Gates P, George R, Miller E. **The effect of underlying clinical conditions on the risk of developing invasive pneumococcal disease in England.** *The journal of Infection*. 2012; **65(1)**:17-24.

2. Rozenbaum MH, van Hoek AJ, Fleming D, Trotter CL, Miller E, Edmunds WJ. **Vaccination of risk groups in England using the 13 valent pneumococcal conjugate vaccine: economic analysis.** *BMJ*. 2012; 345():e6879.

3. Smith KJ, Wateska AR, Nowalk MP, Raymund M, Nuorti JP, Zimmerman RK. **Cost-effectiveness of adult vaccination strategies using pneumococcal conjugate vaccine compared with pneumococcal polysaccharide vaccine**. *JAMA.* 2012; **307(8)**:804-12.

4. Tan SS, Bouwmans CA, Rutten FF, Hakkaart-van Roijen L. **Update of the Dutch Manual for Costing in Economic Evaluations**. *Int J Technol Assess Health Care.* 2012; **28(2)**:152-158.

5. Mangen MJ, Rozenbaum MH, Huijts SM, van Werkhoven CH, Postma DF, Atwood M, et al. **Cost-effectiveness of adult pneumococcal conjugate vaccination in the Netherlands**. *The European respiratory journal.* 2015; ERJ-00325-2015; doi: 10.1183/13993003.00325-2015.
